# Another Rare Cause of Hypertrophic Olivary Degeneration Following Cavernous Malformation Hemorrhage: A Case Report

**DOI:** 10.3390/diagnostics15162048

**Published:** 2025-08-15

**Authors:** Sigita Skrastiņa, Marija Roddate, Kristaps Rancāns, Evija Miglāne, Aleksandrs Kalniņš, Arturs Balodis

**Affiliations:** 1 Faculty of Medicine, Riga Stradins University, 16 Dzirciema Street, LV-1007 Riga, Latvia; sigita.sk14@gmail.com; 2 Clinic of Neurology, Pauls Stradins Clinical University Hospital, 13 Pilsonu Street, LV-1002 Riga, Latvia; m.roddate@gmail.com (M.R.); evija.miglane@rsu.lv (E.M.); 3 Department of Neurosurgery, Pauls Stradins Clinical University Hospital, 13 Pilsonu Street, LV-1002 Riga, Latvia; kristapsrancans@gmail.com; 4 Department of Neurology and Neurosurgery, Riga Stradins University, 16 Dzirciema Street, LV-1007 Riga, Latvia; 5 Division of Neuroradiology, Department of Radiology, Pritzker School of Medicine, The University of Chicago Medical Center, 5841 S. Maryland Avenue, Chicago, IL 60637, USA; alekskalnins@gmail.com; 6 Institute of Diagnostic Radiology, Pauls Stradins Clinical University Hospital, 13 Pilsonu Street, LV-1002 Riga, Latvia; 7 Department of Radiology, Riga Stradins University, 16 Dzirciema Street, LV-1007 Riga, Latvia

**Keywords:** hypertrophic olivary degeneration, magnetic resonance imaging, tractography, cavernous malformation

## Abstract

**Introduction**: Hypertrophic olivary degeneration (HOD) is a rare form of trans-synaptic degeneration involving the Guillain–Mollaret triangle, characterized by enlargement of the inferior olivary nucleus—unlike the atrophy typical of most neurodegenerative processes. It is usually associated with stroke, surgical injury, or demyelination, but rarely follows hemorrhage from a cavernous malformation (CM). This report presents a case of HOD secondary to a mesencephalic CM hemorrhage, with emphasis on imaging findings and diagnostic considerations. **Case Description:** A 55-year-old woman presented with acute-onset, right-sided facial, torso, and limb hypoesthesia, along with gait instability. Neurological examination revealed sensory impairment in the right maxillary (V2) and mandibular (V3) trigeminal territories, as well as diminished pain and temperature sensation throughout the right hemibody. MRI revealed a hemorrhage in the posterior mesencephalon near the left red nucleus, leading to the diagnosis of a CM with an associated venous angioma. She was managed conservatively and improved clinically. Six months later, MRI showed hypertrophy and T2/FLAIR hyperintensity of the left inferior olive, consistent with developing HOD. At 1.5 years follow-up, olivary enlargement had progressed—now consistent with stage 2 HOD—and a bilateral palatal tremor was observed, more pronounced on the right side. DTI revealed asymmetric volume loss in the left brainstem fiber pathways at the level of the medulla oblongata, confirming trans-synaptic degeneration. **Conclusions**: This case highlights HOD as a rare but important complication of mesencephalic CM hemorrhage. Recognition of its characteristic imaging features—olivary hypertrophy with persistent T2/FLAIR hyperintensity—is essential for accurate diagnosis. DTI supports the trans-synaptic mechanism, helping distinguish HOD from other pathologies and preventing unnecessary investigations.

## 1. Background

**Figure 1:**
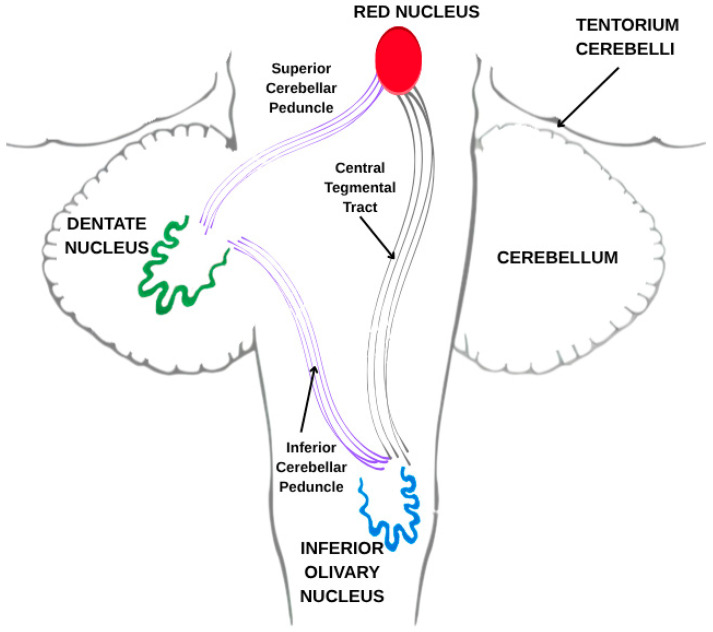
Graphic representation of the dentato–rubro–olivary pathway (triangle of Guillain–Mollaret). Image made by the authors.

Hypertrophic olivary degeneration (HOD) is an uncommon form of trans-synaptic degeneration that affects the dentato–rubro–olivary pathway, also known as the Guillain–Mollaret triangle (Figure 1). This neural circuit includes the red nucleus in the midbrain, the ipsilateral inferior olivary nucleus (ION) in the medulla oblongata, and the contralateral dentate nucleus of the cerebellum, interconnected via the central tegmental tract and superior cerebellar peduncle [1]. Disruption of any component within this triangle—typically as a result of ischemic stroke, hemorrhage, surgical trauma, or demyelinating disease [2,3]—can lead to secondary effects on the ION.

Unlike most neurodegenerative processes, which are characterized by atrophy, HOD is defined by hypertrophy of the ION, often accompanied by vacuolar degeneration, astrocytic gliosis, and neuronal enlargement [4]. This distinctive pathophysiology likely reflects a combination of denervation hypersensitivity, reactive gliosis, and impaired synaptic input, and is often associated with delayed clinical symptoms. These may include palatal tremor, ocular myoclonus, nystagmus, or other signs of brainstem dysfunction, which typically emerge weeks to months following the primary insult [4].

MRI plays a central role in the identification and staging of HOD. The hallmark imaging features include T2-weighted and FLAIR hyperintensity within the ION, frequently accompanied by olivary enlargement in the earlier stages [5]. These findings evolve in a predictable temporal pattern, which has been categorized into a widely used three-stage MRI classification system (Table 1) [6,7]. This staging system reflects both the imaging progression and underlying histopathological changes, thereby providing a framework for interpreting longitudinal radiological findings in affected patients.

While HOD has been most reported in the setting of pontine infarction or cerebellar hemorrhage, its development following hemorrhage from a cavernous malformation (CM)—particularly in the mesencephalon—is rare and sparsely documented in the literature [1,3,8,9,10,11]. CMs are vascular lesions composed of dilated, thin-walled capillary channels without intervening brain parenchyma. When located within the brainstem, they pose a high risk for neurological sequelae due to the compact arrangement of critical tracts and nuclei. Hemorrhage from a mesencephalic CM has the potential to damage the central tegmental tract, thereby predisposing to the development of HOD.

Given the often-delayed onset of symptoms and the non-specific nature of olivary signal changes, recognition of HOD requires a high index of suspicion [7,12]. Without appropriate clinical context, the characteristic imaging findings may be misinterpreted as representing neoplastic, inflammatory, or infectious pathology. Moreover, the presence of olivary hypertrophy—rather than atrophy—can lead to further diagnostic uncertainty. Awareness of the temporal radiological evolution of HOD and familiarity with its MRI classification are therefore essential for accurate diagnosis and to prevent unnecessary investigations.

This case report presents the radiological and clinical evolution of HOD in a patient with a hemorrhage in the posterior mesencephalon near the left red nucleus, highlighting the characteristic imaging features, tractographic evidence of trans-synaptic degeneration, and the relevance of staged MRI interpretation in clinical practice.

## 2. Case Report

A 53-year-old woman with no prior neurological history presented in December 2023 with acute-onset, right-sided sensory disturbances, including facial, torso, and limb hypoesthesia, accompanied by gait instability. Neurological examination revealed reduced sensation in the maxillary (V2) and mandibular (V3) territories of the right trigeminal nerve, as well as diminished pain, temperature, and light touch sensation across the right hemibody. Coordination testing demonstrated dysmetria of the right upper limb, and Romberg’s test showed instability with a tendency to fall to the right. Horizontal binocular nystagmus was evident during leftward gaze, and gait testing demonstrated Romberg instability. The patient remained alert and oriented, with a Glasgow Coma Scale (GCS) score of 15, a National Institutes of Health Stroke Scale (NIHSS) score of 2, and a modified Rankin Scale (mRS) score of 2.

**Figure 2:**
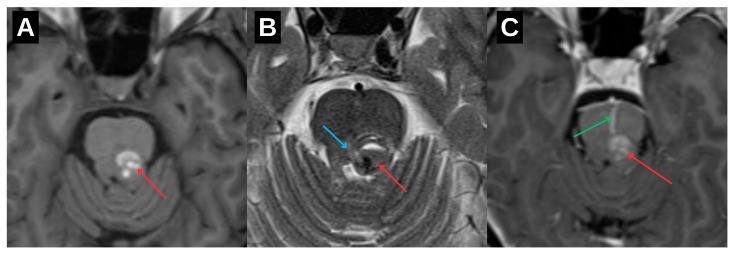
Brain MRI on axial T1-weighted (**A**), T2-weighted (**B**), and contrast-enhanced T1-weighted (**C**) sequences reveal a cavernoma centered in the left tegmentum of the mesencephalon (red arrow). The lesion demonstrates both extracellular and intracellular methemoglobin, indicating subacute hemorrhage. Mild surrounding edema appears slightly hyperintense on T2-weighted images and is associated with a small fluid–fluid level (blue arrow). An associated venous angioma is visualized (green arrow), showing prominent contrast enhancement.

**Figure 3:**
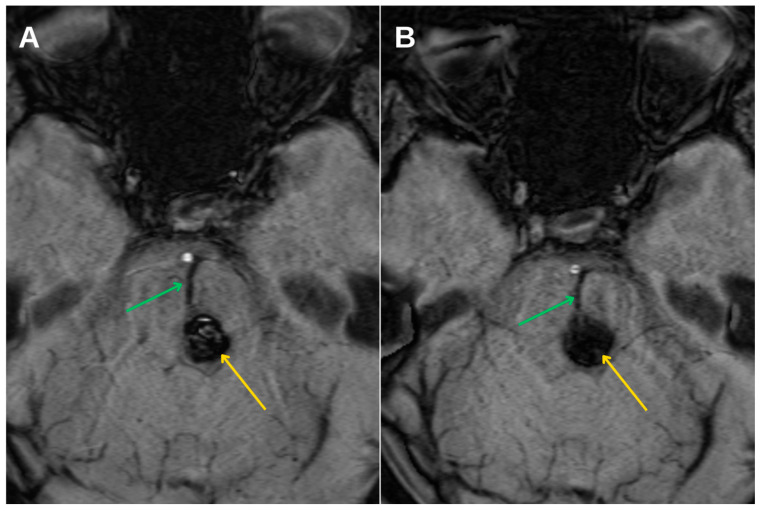
Axial SWI images of the mesencephalon at initial presentation (**A**) and at 1.5 years follow-up (**B**). Image (**A**) demonstrates blooming artifact consistent with acute hemorrhage from a cavernous malformation (yellow arrow) in the posterior mesencephalon near the left red nucleus, along with a ventrally extending associated venous angioma (green arrow). Image (**B**) shows lesion stabilization with persistent hemosiderin deposition and continued visualization of the venous angioma.

Initial neuroimaging with non-contrast computed tomography followed by magnetic resonance imaging (MRI) revealed a hemorrhagic lesion located in the dorsal portion of the upper brainstem, involving the left mesencephalon and extending toward the pontomesencephalic junction (Figure 2). The lesion demonstrated imaging characteristics typical of a cavernous malformation, accompanied by a venous angioma (Figure 3). Given the absence of acute hydrocephalus, mass effect, or clinical deterioration, a conservative therapeutic approach was adopted. The patient was managed with intravenous corticosteroids and osmotic agents to reduce perilesional edema and was initiated on symptomatic treatment with gabapentinoids to address neuropathic symptoms. No surgical intervention was indicated at that time, and the patient exhibited gradual clinical improvement.

Subsequent imaging follow-up after three months demonstrated expected interval evolution of the hemorrhagic component, with partial resorption of blood products and reduced surrounding edema.

However, the patient continued to report intermittent paresthesia involving the right limbs and perioral region, suggesting persistent dysfunction along brainstem sensory pathways. A high-resolution follow-up MRI with contrast was performed six months after the initial episode, including T2-weighted, FLAIR and susceptibility-weighted sequences, to evaluate for delayed complications. The imaging study demonstrated a new finding of hypertrophy and T2/FLAIR hyperintensity involving the left inferior olivary nucleus. These changes were not present on initial imaging and were interpreted as consistent with evolving hypertrophic olivary degeneration (HOD). The underlying cavernous malformation remained stable in size, without signs of rebleeding, and no new lesions were identified elsewhere in the brainstem. The absence of mass effect, diffusion restriction, or contrast enhancement excluded alternative pathologies such as neoplasm, infection, or demyelination [13,14].

**Figure 4:**
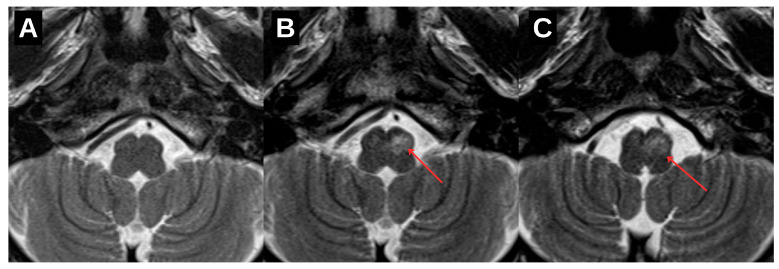
Axial T2-weighted MRI images in dynamics demonstrating hypertrophic olivary degeneration (HOD) on the left side. (**A**) Initial MRI at the time of hemorrhage in December 2023 reveals no changes in the inferior olivary nucleus. (**B**) Follow-up MRI 6 months later shows early hypertrophy and hyperintensity of the left inferior olivary nucleus (red arrow), consistent with developing HOD. (**C**) MRI at 1.5 years post-hemorrhage demonstrates further enlargement of the nucleus with persistent FLAIR hyperintensity, corresponding to stage 3 of HOD.

Further progression of olivary changes was noted on repeat MRI performed 1.5 years after the initial hemorrhagic event, which demonstrated continued enlargement of the inferior olivary nucleus, with the craniocaudal diameter reaching approximately 1.6 cm and anteroposterior thickness of 0.5 cm. Signal abnormalities on T2-weighted and FLAIR sequences persisted, while no atrophy was observed (Figure 4C). These radiological findings were consistent with Stage 2 in the traditional three-stage classification of HOD, representing the hypertrophic phase.

**Figure 5:**
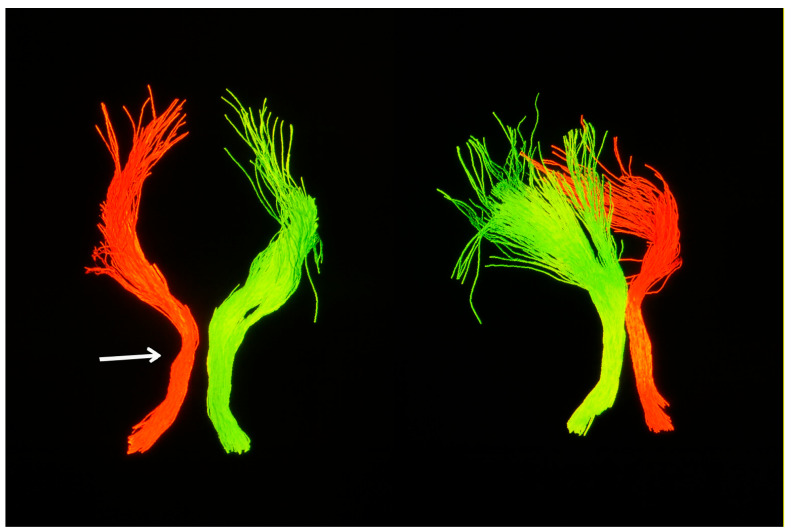
Diffusion tensor imaging (DTI) tractography illustrating brainstem fiber architecture at the level of the medulla oblongata (white arrow). The left-sided tract bundle (red) demonstrates reduced volume compared to the contralateral side (green), suggestive of asymmetric white matter integrity loss. While individual tracts such as the central tegmental tract cannot be distinctly delineated, the observed volume reduction—when interpreted in conjunction with conventional MRI findings—is consistent with trans-synaptic degeneration within the dentato–rubro–olivary pathway.

Clinically, the patient remained functionally stable with no deterioration in higher cortical function or motor strength. However, for the first time at the 1.5-year follow-up, a palatal tremor was identified on examination—an involuntary, rhythmic movement of the soft palate that was bilateral, but more pronounced on the right side—classically associated with interruption of the dentato–rubro–olivary pathway (See Video S1). This clinical sign further supported involvement of the Guillain–Mollaret triangle. Complementary diffusion tensor imaging (DTI) revealed partial disruption and reduced fiber integrity in the left central tegmental tract, confirming the presence of trans-synaptic degeneration originating from the prior brainstem hemorrhage (Figure 5).

DTI was performed using a 1.5 T Siemens MAGNETOM Sola system with a single-shot echo-planar imaging (EPI) sequence optimized for brainstem tractography. Post-processing and fiber tracking were performed using StealthStation™ S8 planning software (Medtronic), applying deterministic tractography with a minimum fiber length threshold of 10 mm and angular threshold of 45° to suppress spurious fibers and preserve anatomical plausibility. Regions of interest were manually defined in the medulla oblongata to evaluate the integrity of the brainstem white matter tracts.

When considered in combination, the clinical and radiological findings established a diagnosis of hypertrophic olivary degeneration as a delayed complication of hemorrhage from a cavernous malformation in the posterior mesencephalon near the red nucleus. The temporal evolution, characteristic MRI features, and tractography abnormalities illustrated the classic progression of HOD and highlighted the importance of recognizing this entity in the context of delayed-onset neurological symptoms following brainstem injury.

## 3. Discussion

**Figure 6:**
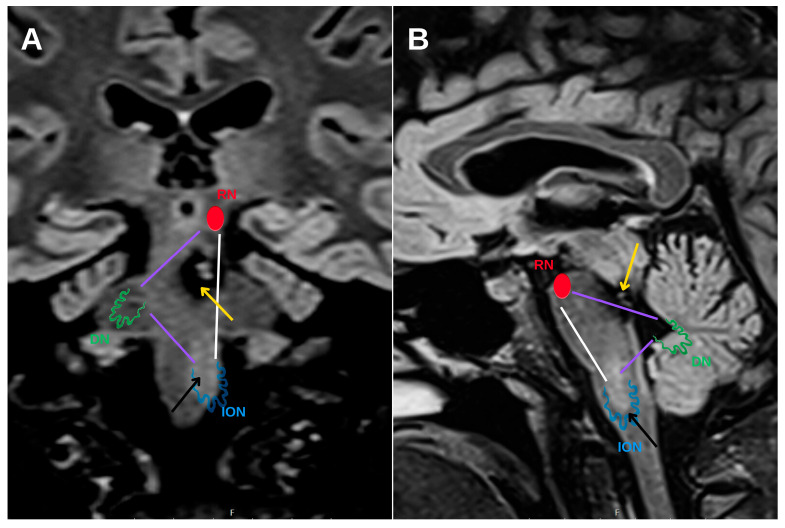
Cerebellar MRI on coronal (**A**) and sagittal (**B**) FLAIR sequences (**A**) with a schematic overlay of the Guillain–Mollaret triangle (DN: dentate nucleus; RN: red nucleus; ION: inferior olivary nucleus). The purple lines represent the connections between nuclei, and the white line represents the central tegmental tract. A mesencephalic cavernoma, exhibiting chronic hemorrhagic changes with hemosiderin deposition, is evident in both the coronal and sagittal sections (yellow arrows), while the black arrow indicates the affected inferior olivary nucleus.

HOD represents a distinct form of trans-synaptic degeneration characterized by hypertrophy rather than atrophy of the ION, most frequently resulting from disruption of the dentato–rubro–olivary pathway, commonly referred to as the Guillain–Mollaret triangle [1]. This anatomical circuit comprises the contralateral dentate nucleus, the ipsilateral red nucleus, and the ipsilateral ION, interconnected via the superior cerebellar peduncle and the central tegmental tract. Lesions affecting any component of this circuit—particularly the central tegmental tract—may lead to secondary degeneration of the ION (Figure 6).

While the majority of HOD cases described in the literature are associated with ischemic stroke, surgical trauma, or demyelinating disease affecting the pons or cerebellum [2,3], development of HOD following hemorrhage due to a mesencephalic cavernous malformation (CM) is exceedingly rare [1]. In particular, spontaneous (non-surgical) hemorrhage in the posterior mesencephalon—adjacent to the red nucleus—is an uncommon etiology.

Compared to previously reported cases, this report highlights several distinguishing features. First, the lesion location in the posterior mesencephalon near the left red nucleus differs from the more typical pontine or cerebellar cases. Secondly, we present longitudinal MRI follow-up over a 1.5-year period, showing the progression from early hypertrophic changes to the development of a bilateral palatal tremor—a hallmark of delayed clinical manifestation. This finding can also be considered a novel aspect of our study, as similar cases supported by illustrative video documentation are rarely described in the literature. Finally, the inclusion of DTI, which demonstrated asymmetric disruption of brainstem tracts, adds anatomical and diagnostic depth. Although DTI is not routinely applied in HOD assessment, its use here supports the trans-synaptic mechanism and enhances radiological interpretation.

To further underscore the distinctive features of our case, we compiled a comparative summary (Table 2) of previously reported instances of hypertrophic olivary degeneration (HOD) following brainstem cavernous malformations. This overview emphasizes key differences across studies in terms of lesion location, etiology (spontaneous vs. post-surgical), laterality of HOD, use of diffusion tensor imaging, and clinical presentation, particularly the presence or absence of palatal tremor. To identify relevant cases, we performed a structured literature search using the PubMed database with the keywords “hypertrophic olivary degeneration” and “cavernous malformation”. Our case uniquely combines spontaneous mesencephalic hemorrhage, progressive radiological evolution, symptomatic bilateral palatal tremor, and DTI-confirmed tract disruption—a combination rarely detailed in the literature.

The pathophysiological hallmark of HOD lies in its paradoxical hypertrophic response. Histologically, it is characterized by neuronal vacuolization, astrocytic gliosis, and neuronal enlargement. The underlying mechanism is thought to involve a combination of trans-synaptic degeneration, disinhibition, and denervation hypersensitivity. Clinically, HOD may manifest with delayed-onset neurological signs, including palatal tremor, ocular myoclonus, and nystagmus [4]. In the current case, palatal tremor became apparent 18 months after the initial hemorrhage, during the established hypertrophic phase (stage 2) of the classical MRI-based staging system, characterized by persistent olivary enlargement and T2/FLAIR hyperintensity.

Magnetic resonance imaging (MRI) remains the cornerstone of HOD diagnosis and staging. The condition demonstrates a predictable radiological progression: T2-weighted and FLAIR hyperintensity of the ION without hypertrophy in the early phase (stage 1), evolving into hypertrophy with persistent signal hyperintensity (stage 2), and eventually normalization or mild atrophy with persistent hyperintensity (stage 3). In this patient, serial MRI examinations at 6 months and again at 1.5 years post-hemorrhage demonstrated the expected imaging evolution. This case also provides a rare opportunity to illustrate the dynamic radiological progression of HOD over time, as visualized in Figure 4. Such longitudinal documentation is uncommon in the literature, underscoring the value of systematic follow-up. Crucially, the absence of mass effect, contrast enhancement, or diffusion restriction helped exclude neoplastic, infectious, or demyelinating etiologies [14].

An important adjunct in this case was diffusion tensor imaging (DTI), which demonstrated partial disruption of the left central tegmental tract. DTI-based tractography, while not routinely employed in clinical practice, offers unique value in delineating microstructural integrity and confirming trans-synaptic degeneration along the dentato–rubro–olivary pathway [5]. In this context, it served to anatomically validate the presumed mechanism underlying the observed radiological and clinical findings.

Given the high concentration of critical neural tracts in the brainstem, cavernous malformations (CMs) in this location warrant meticulous long-term clinical and radiological follow-up. Although many brainstem CMs remain asymptomatic or present with subtle symptoms, they are associated with a significantly higher annual risk of hemorrhage compared to supratentorial lesions—particularly following an initial bleeding event. Even minor or clinically silent hemorrhages may result in irreversible damage to adjacent white matter pathways, potentially leading to delayed complications such as hypertrophic olivary degeneration. This underscores the importance of serial high-resolution MRI, not only to monitor lesion stability and detect signs of rebleeding, but also to identify secondary neurodegenerative changes in a timely manner. Early recognition of HOD is clinically important as it may prevent misdiagnosis and unnecessary investigations, particularly when patients present with delayed-onset brainstem symptoms such as tremor, ataxia, or dysarthria. Awareness of the characteristic MRI evolution and typical lesion location helps differentiate HOD from neoplastic, demyelinating, or infectious processes. Although HOD itself is not reversible, timely diagnosis can guide appropriate symptom management and allow for targeted neurological rehabilitation. From a prognostic standpoint, the presence of HOD may indicate prior irreversible disruption of the dentato–rubro–olivary pathway, which in turn suggests a higher likelihood of persistent motor symptoms, including palatal tremor. Follow-up strategies should be individualized based on lesion size, location, prior hemorrhagic history, and evolving symptomatology, with a low threshold for repeat imaging in the presence of new or unexplained neurological signs.

While no disease-modifying treatments exist for hypertrophic olivary degeneration, symptomatic management of palatal tremor may be considered. Pharmacological options include clonazepam, valproate, gabapentin, flunarizine, lamotrigine, trihexyphenidyl, and piracetam, among others, though response is often variable [22]. From Refs. [4,22,23], in select cases botulinum toxin injections into the tensor veli palatini or levator veli palatini muscles have been shown to reduce tremor severity and associated auditory symptoms. From Ref. [24], non-pharmacological approaches such as radiofrequency ablation and cognitive behavioral therapy have also been described in rare contexts. As presented in Refs. [4,25], stereotactic procedures may be considered in drug-refractory cases. In the present case, no specific treatment for the tremor was initiated, given its limited functional impact. Nevertheless, the emergence of new neurological signs long after the initial hemorrhagic event underscores the importance of long-term clinical and radiological surveillance in patients with brainstem CMs, even in the absence of rebleeding or overt progression of the primary lesion.

Currently, there are no disease-modifying therapies for HOD. Management is symptomatic, and interventions such as gabapentinoids, anticholinergics, or botulinum toxin have shown variable efficacy in treating palatal tremor.

In summary, this case illustrates the diagnostic significance of recognizing HOD as a delayed complication of brainstem cavernoma hemorrhage. It highlights the role of high-resolution MRI and advanced neuroimaging techniques such as DTI in confirming diagnosis and understanding pathophysiology. Increased awareness of this entity may help prevent misdiagnosis and unnecessary investigations in patients presenting with delayed-onset brainstem symptoms.

## 4. Conclusions

HOD is another rare and frequently underrecognized complication of brainstem injury, most commonly associated with disruption of the Guillain–Mollaret triangle. Recognition of its characteristic MRI features—particularly olivary hypertrophy accompanied by persistent T2/FLAIR hyperintensity—is essential for accurate diagnosis. This case demonstrates that hemorrhage from a cavernous malformation in the posterior mesencephalon can result in delayed trans-synaptic degeneration of the inferior olivary nucleus, with a typical temporal evolution on MRI and clinical manifestation of palatal tremor.

The findings underscore the importance of long-term clinical and radiological monitoring in patients with brainstem cavernomas, even when initial symptoms appear to be resolved. Future studies may further elucidate the role of tractography in predicting and characterizing the progression of HOD, as well as the potential benefits of targeted symptomatic therapies, particularly in patients who develop tremor or other signs of brainstem dysfunction.

## Figures and Tables

**Table 1:** Three-stage MRI classification of hypertrophic olivary degeneration [6,7].

Stage	Time After Injury	MRI Findings	Histopathological Changes
Stage 1 (Pre-hypertrophic phase)	1 month to 6 months	–Increased signal on T2-weighted and proton density (PD) images. –No hypertrophy of the inferior olivary nucleus (ION).	–Early trans-synaptic degeneration. –Initial neuronal loss. –Gliosis begins.
Stage 2 (Hypertrophic phase)	6 months to 3–4 years	–Persistent T2/PD hyperintensity. –Hypertrophy of the ION.	–Prominent vacuolar degeneration. –Neuronal dissolution. –Marked astrocytic gliosis. –Enlargement of olivary neurons and surrounding neuropil.
Stage 3 (Post-hypertrophic/chronic phase)	From 3 to 4 years onward (indefinite)	–Persistent T2/PD hyperintensity. –Resolution of hypertrophy (may normalize or become mildly atrophic).	–Residual gliosis. –Loss of hypertrophy. –Possible mild atrophy of the ION.

**Table 2:** Comparative overview of previous cases of HOD that developed after the brainstem cavernous malformation.

Author (Year)	Age/Sex	Etiology	Lesion/CM Site	HOD Site	DTI Used	Palatal Tremor
Asal et al. [9] (2011)	36/M	Spontaneous CM hemorrhage	Ponto-mesencephalic	Bilateral	No	No
Carvalho et al. [15] (2015)	60/F	Post-surgical CM resection	Tectal plate	L	Yes	Yes
Dogan et al. [11] (2020)	62/M	Spontaneous CM hemorrhage	Pontine/R	R	No	No
Gatlin et al. [16] (2011)	35/M	Post-surgical CM resection	Pontine/L	L	No	No
Harter and Davis [17] (2004)	32/M	Post-surgical CM resection	Ponto-mesencephalic/L	L	No	No
Hornyak et al. [18] (2008)	44/M	Post-surgical CM resection	Pontine/L	L	No	Yes
Hornyak et al. [18] (2008)	50/F	Post-surgical CM resection	Pontine/L	L	No	No
Hornyak et al. [18] (2008)	53/M	Post-surgical CM resection	Ponto-mesencephalic/Midline	Bilateral	No	No
Hornyak et al. [18] (2008)	47/F	Post-surgical CM resection	Mesencephalic/L	Bilateral	No	Yes
Houssni et al. [13] (2024)	26/M	Infratentorial cavernomatosis, orbital cavernous hemangioma	Cerebellar/L	Bilateral	No	Yes
Howard et al. [19] (2019)	58/M	Post-surgical CM resection	Pontine/L	L	No	No
Kosaka et al. [20] (2014)	53/M	-	Central tegmental/R	R	No	No
Macht et al. [8] (2009)	52/F	Spontaneous CM hemorrhage	Ponto-mesencephalic/R	R	No	No
Wein et al. [10] (2015)	58/M	Spontaneous CM hemorrhage	Pontine/R	R	No	No
Zhu et al. [21] (2024)	34/M	Post-surgical CM resection	Ponto-mesencephalic/R	Bilateral	No	No
This report (2025)	55 y/F	Spontaneous CM hemorrhage	Mesencephalic/L	L	Yes	Yes

M–male, F–female, L–left, R–right, CM–cavernous malformation.

## Data Availability

The data presented in this study are available on request from the corresponding author.

## Supplementary Materials

The following supporting information can be downloaded at: https://www.mdpi.com/article/10.3390/diagnostics15162048/s1, Video S1: Palatal Tremor in Hypertrophic Olivary Degeneration.

## Abbreviations

The following abbreviations are used in this manuscript:

HOD	Hypertrophic olivary degeneration
ION	Inferior olivary nucleus
MRI	Magnetic resonance imaging
FLAIR	Fluid-attenuated inversion recovery
PD	Proton density
CM	Cavernous malformation
DTI	Diffusion tensor imaging
CT	Computed tomography
GCS	Glasgow Coma Scale
NIHSS	National Institutes of Health Stroke Scale
mMRS	Modified Rankin Scale
